# Knee adduction moment decomposition: Toward better clinical decision-making

**DOI:** 10.3389/fbioe.2022.1017711

**Published:** 2022-11-18

**Authors:** Mina Baniasad, Robin Martin, Xavier Crevoisier, Claude Pichonnaz, Fabio Becce, Kamiar Aminian

**Affiliations:** ^1^ Laboratory of Movement Analysis and Measurement, Ecole Polytechnique Fédérale de Lausanne, Lausanne, Switzerland; ^2^ Department of Orthopaedic Surgery and Traumatology, Lausanne University Hospital and University of Lausanne, Lausanne, Switzerland; ^3^ Department of Physiotherapy, School of Health Sciences HESAV, HES-SO University of Applied Sciences and Arts Western Switzerland, Lausanne, Switzerland; ^4^ Department of Diagnostic and Interventional Radiology, Lausanne University Hospital and University of Lausanne, Lausanne, Switzerland

**Keywords:** gait, ground reaction vector, knee adduction moment, osteoarthritis, personalized treatment, kinetics, inverse dynamics

## Abstract

Knee adduction moment (KAM) is correlated with the progression of medial knee osteoarthritis (OA). Although a generic gait modification can reduce the KAM in some patients, it may have a reverse effect on other patients. We proposed the “decomposed ground reaction vector” (dGRV) model to 1) distinguish between the components of the KAM and their contribution to the first and second peaks and KAM impulse and 2) examine how medial knee OA, gait speed, and a brace influence these components. Using inverse dynamics as the reference, we calculated the KAM of 12 healthy participants and 12 patients with varus deformity and medial knee OA walking with/without a brace and at three speeds. The dGRV model divided the KAM into four components defined by the ground reaction force (GRF) and associated lever arms described with biomechanical factors related to gait modifications. The dGRV model predicted the KAM profile with a coefficient of multiple correlations of 0.98 ± 0.01. The main cause of increased KAM in the medial knee OA group, the second component (generated by the vertical GRF and mediolateral distance between the knee and ankle joint centers), was decreased by the brace in the healthy group. The first peak increased, and KAM impulse decreased with increasing velocity in both groups, while no significant change was observed in the second peak. The four-component dGRV model successfully estimated the KAM in all tested conditions. It explains why similar gait modifications produce different KAM reductions in subjects. Thus, more personalized gait rehabilitation, targeting elevated components, can be considered.

## Introduction

Knee osteoarthritis (OA) is one of the leading causes of disability in the aged population (Murray et al., 2012). The first peak of the double-bump pattern of the KAM during stance correlates with the presence (Hurwitz et al., 2002), severity (Foroughi et al., 2009), and pain (L.E. et al., 2007) in knee OA. Typically, the 3D knee joint moments, including KAM, are estimated by inverse dynamics (ID) (Baker et al., 2018), considering inertial, gravitational, and ground reaction terms. The third term, that is, the ground reaction contribution, is the cross product of the ground reaction force (GRF) and the vector connecting the joint center to the center of pressure (COP). By neglecting the contribution of the inertial and gravitational terms that reduce the computational cost, the ground reaction vector (GRV) alone has been used for estimation of the KAM (Wells, 1981; Fantozzi et al., 2012) with a root mean square error of 4% in the first peak (Baniasad et al., 2020). Implementing GRV for KAM estimation requires the vector connecting the COP to the knee joint center and is typically computed through 3D positions of the markers, regardless of how much of that is due to the COP position, foot orientation, or tibia inclination. For example, the lever arm in the GRV method could be the same for two patients, one with the very internal position of COP and normal tibia varus angle and the other with a lateral COP position but varus orientation of tibia. Although in view of GRV, they have similar lever arms and similar KAM, a treatment aiming to decrease their KAM based on gait modifications should be designed in entirely different ways. For the first patient, a proper insole might be appropriate, while the second patient may benefit from the valgus bracing (Shelburne et al., 2008). Despite its simplicity, the GRV does not reflect a clear vision of the relationship between gait modification parameters and the KAM.

Gait modifications such as alterations in foot progression angle (FPA) (Wang et al., 2021), speed (Robbins and Maly, 2009), step width (Xu et al., 2020; Pereira et al., 2021), medial thrust (Ferrigno et al., 2016), and non-invasive intervention such as unloader braces (Moyer et al., 2015) and lateral wedge insoles (Xing et al., 2017) have been proposed to decrease the KAM. However, controversies about the KAM reduction have been raised in different studies (Jenkyn et al., 2008; Wang et al., 2021): using a similar method and using a uniform method within one study could increase the KAM in some participants (Gerbrands et al., 2014; Uhlrich et al., 2018; Uhlrich, 2020). Tailored intervention and personalized gait modifications (Uhlrich et al., 2018; Uhlrich, 2020) are effective ways to increase the achievable reduction in KAM (Richards et al., 2017).

Personalizing gait modifications is mainly performed through real-time biofeedback (Cheung et al., 2018), using an instrumented treadmill and a trial-and-error method. However, though this method clinically confirms that various components may influence the generic KAM, it does not allow to anticipate which correction suits the patient’s need or to differentiate study subgroups within those who have increased KAM. Understanding the sources of the KAM by a proper decomposition would help categorize patients and set suitable gait modifications to target the increased components.

In this study, our primary aim was to evaluate the concurrent validity of a model that decomposes the KAM into components. These components should be described with understandable biomechanical factors used in gait modifications. To do so, we developed the decomposed GRV (dGRV) model and investigated the contribution of each component to three main KAM features, that is, first peak, second peak, and KAM impulse. This model could show the percentage of KAM generated by parameters that are altered in gait modifications, such as toe-in/toe-out, varus orientation of tibia (altered by valgus bracing or medial thrust), the mediolateral position of COP (lateralized by proper insole), or mediolateral component of GRF (controlled by lateral trunk movement). When investigating the effect of a gait modification instead of the general KAM, the comparison can be made at the level of these components to realize the intervention mechanism and better understand which components are decreased as a result. We hypothesized that the GRV method is sufficiently accurate and reliable as a basis for our proposed model. Our secondary aim was to show an application of this model by investigating how KAM components alter in patients with varus deformity and medial knee OA compared to healthy volunteers, and how an ankle–foot orthosis known as a knee unloader brace (Menger et al., 2016) affects the KAM components in healthy volunteers. We hypothesized that the brace decreases the KAM by reducing the component related to tibia varus orientation, which causes increased KAM in patients with varus deformity. This study is clinically important because the component-level comparisons can help determine which component causes increased KAM in a patient. This would enable more personalized and thus more effective treatment planning in comparison to the current best practices in which general methods are implemented that may not be effective for every patient.

## Materials and methods

### Participants

This cross-sectional observational study was approved by the institutional ethics committee (CER-VD protocol 2020-01894). A total of 12 patients with varus deformity and medial knee OA and 12 healthy volunteers were enrolled after giving informed consent. The inclusion criteria for the medial knee OA group were 1) diagnosed with symptomatic knee OA with a varus deformity defined as a hip–knee–ankle angle of more than 3 degrees of neutral on standing hip-to-ankle radiograph (EOS imaging, Paris, France), planned for medial open wedge high tibial osteotomy; 2) the ability to walk safely for 30 min without aids; and 3) body mass index less than 35 kg/m^2^. Healthy volunteers were recruited if 1) they presented no pain or history of injury or surgery in the lower extremities; 2) less than 3 degrees of varus/valgus malalignment (hip–knee–ankle angle deviated from 180 degrees) on standing radiograph; 3) body mass index less than 35 kg/m^2^; and 4) age between 18 and 50 years old. In the healthy group, both knees were analyzed, whereas in the patient group, both knees were included only if OA was bilateral. Table 1 summarizes the demographic data of all participants. The Knee injury and Osteoarthritis Outcome Score (KOOS) (Ornetti et al., 2008) was collected for the patients with varus deformity and medial knee OA during their regular preoperative visit with the orthopedic surgeon.

**TABLE 1:** Demographics of healthy and patient groups. Data are presented as mean ± SD. The Knee Injury and Osteoarthritis Outcome Score (KOOS) (Ornetti et al., 2008).

	Healthy (N = 12)	Patients (N = 12)
Age (yrs)	34.3 ± 9.5	48.9 ± 11.6
Height (cm)	177.5 ± 6.5	174.7 ± 11.45
Mass (kg)	77.3 ± 16.1	91.5 ± 14.2
BMI (kg/m^2^)	24.7 ± 4.1	29.9 ± 4.96
Sex	11 males, 1 female	10 males, 2 females
Hip knee angle on EOS	0.2 ± 0.5	6.0 ± 2.9
KOOS pain	—	51 ± 18
KOOS activities of daily living (ADL)	—	65 ± 16
KOOS symptoms	—	27 ± 20

### Experimental protocol

**FIGURE 1:**
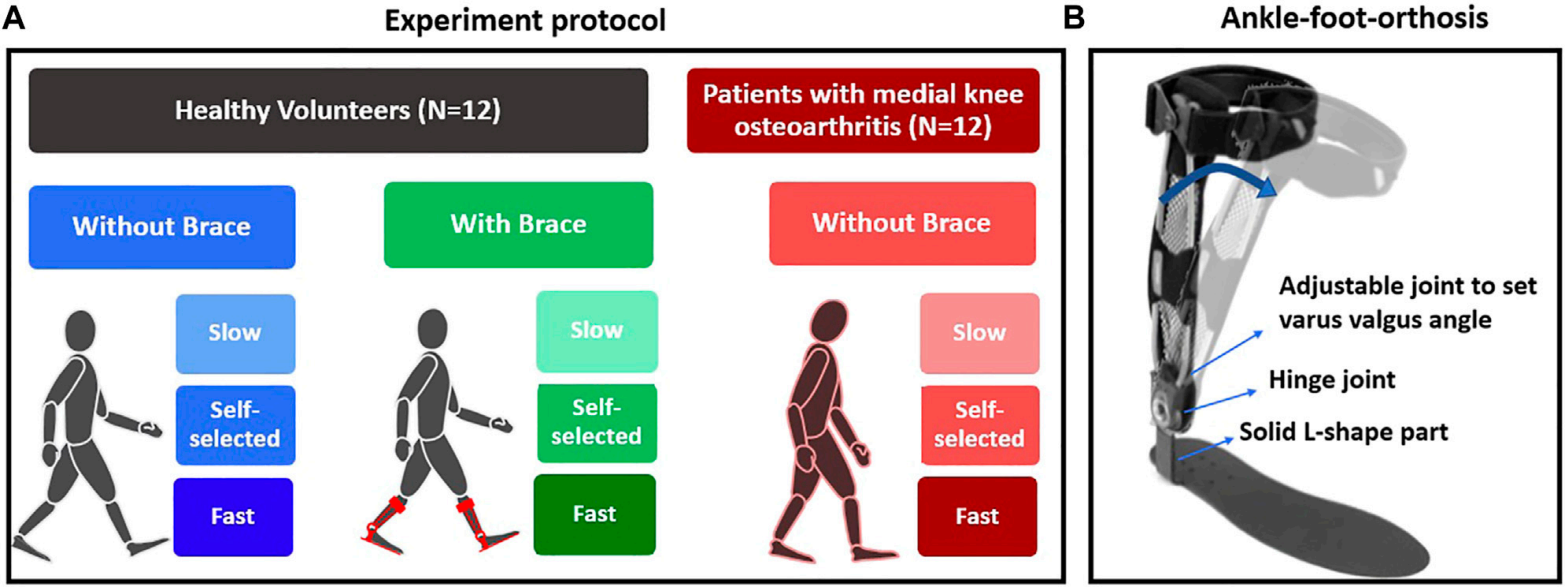
**(A)** Experiment protocol for healthy and patient groups and **(B)** the Agilium Freestep brace used to alter the KAM in the healthy volunteer group.

Gait analysis was performed using a 13-camera Vicon system (Vicon, Oxford Metrics, United Kingdom) along a ten-meter walkway equipped with three ground-embedded force plates (Kistler, Switzerland). Retroreflective markers were attached based on a conventional gait model (Baker et al., 2018). All kinematics and kinetics data were synchronized and collected at 100 Hz and 1,000 Hz, respectively. Participants first completed a static trial with a customized knee alignment device to estimate the knee flexion axis (Baker et al., 2018) and then walked at a self-selected, slow, and fast speed (Figure 1A). To modify the gait in healthy volunteers, they wore the ankle–foot-orthosis (Agilium Freestep, Ottobock, Germany) that pushed the knees medially (Figure 1B). For adaptation, they walked with the brace for several minutes until they felt comfortable, and their stride length became steady, which was observed by marks on the ground. Then, we asked them to walk at a self-selected speed, slow and fast with the brace. We selected this brace because 1) it decreases the KAM (Menger et al., 2016; Petersen et al., 2019); 2) we could control the level of intervention equally for all healthy subjects by setting the angle at 6 degrees, which is the maximum of the brace (compared to alterations in FPA that requires the subject to learn to adjust it and finally could be different between subjects); 3) it requires only a couple of minutes to adapt; and 4) it is not bulky and has minimum interference with markers. For each limb at each condition of walking, we asked them to continue walking until ten successful gait cycles with correct positioning of the feet over the force plates were collected for each side.

### Data analysis

Ground reaction forces and marker positions were low-pass filtered at 8 Hz using a fourth-order Butterworth recursively. The reference KAM was calculated using ID with the Newton–Euler equations (Baker et al., 2018). The reported KAM is expressed in the global reference frame and normalized to percent bodyweight and height (%Bw*Ht). The first and second peaks of the KAM were defined as the maximum value of the KAM profile in the first and second halves of the stance phase, respectively. The angular impulse was computed as the area under the KAM profile. These three features were used to describe the KAM during the stance phase of gait (Chehab et al., 2014). The FPA was calculated as the angle between the axis of the foot in midstance and walking direction (Shull et al., 2011), with toe-out as positive.

**FIGURE 2:**
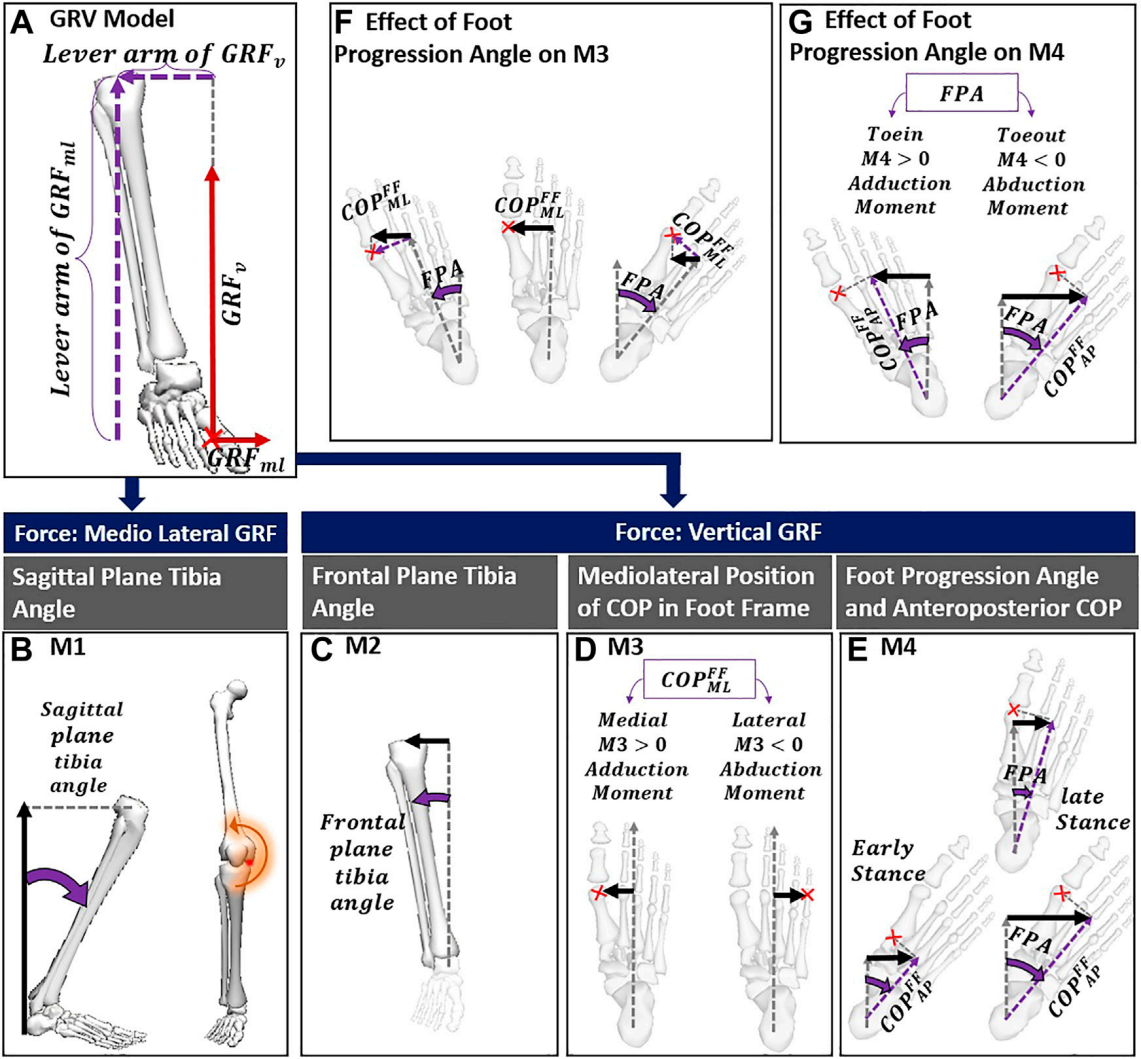
**(A)** GRV model; **(B)** the first component of the dGRV model sourced from mediolateral GRF and knee height; **(C)** the M2 formed by vertical GRF and inclination of tibia in the frontal plane; **(D)** the lever arm of the M3 that can be negative with COP lateral to foot axis; **(E)** the lever arm of M4 can be affected by COP progression from early to late stance with the same FPA, and with variation of FPA; **(F)** with the same position of the COP in the foot frame, it indicates how toe-in/toe-out can reduce the lever arm of M3 while not affecting its sign; **(G)** shows how toe-out/toe-in can reverse the contribution of M4 from adduction to abduction. The red cross indicates the position of the COP. The lever arm of each component is shown with a black arrow. The parameters related to the definition of the lever arm are shown in purple. COP, center of pressure; FPA, foot progression angle; GRV, ground reaction vector; FF, foot frame; AP, anteroposterior; ML, mediolateral.

We proposed a new model (dGRV) that decomposes the GRV estimate of KAM into four components, each moment reflecting a biomechanical aspect of gait adaptation. Starting from the GRV model (Figure 2A), KAM can be expressed in the global frame (GF) using the mediolateral (ML) and vertical (V) components of GRV and knee joint center (KJC) and the mediolateral position of COP ( ${COP}_{ML}^{GF}$ ) as:

$${KAM\;}^{GF}=\overset{︷}{{GRF}_{ML}^{GF}*{KJC}_{V}^{GF}}M1+{GRF}_{V}^{GF}*\left({COP}_{ML}^{GF}-{KJC}_{ML}^{GF}\right)$$ (1)

In the dGRV model, we defined four moments regarding the GRF and different lever arms in the frontal plane. The GRF can cause an external adduction moment on the knee through vertical and mediolateral components. Depending on the lever arm, each force can lead to various levels of the external KAM. From Eq. 1, the first term expresses the effect of mediolateral GRF and constitutes the first component (M1) with the lever arm of the knee height, which corresponds to the sum of the ankle height and vertical projection of shank length ( $L_{Shank}$ ) (Eq. 2; Figure 2B).

$${M1=GRF}_{ML}^{GF}*{KJC}_{V}^{GF}={GRF}_{ML}^{GF}*\left(L_{Shank}*\cos\left(SPTA\right)+AnkleHeight\right)$$, (2)

with SPTA as the sagittal plane tibia angle.

The second term of Eq. 1 is the vertical GRF with the lever arm formed by the mediolateral distance between the knee joint center and COP in GF. Using the ML component of the ankle joint center ( ${AJC}_{ML}^{GF}$ ), we decomposed this distance to more understandable and clinically relevant vectors to provide a more detailed decomposition of this second term as:

$${GRF}_{V}^{GF}*\left({{COP}_{ML}^{GF}-KJC}_{ML}^{GF}\right)=\overset{︷}{{GRF}_{V}^{GF}*\left({AJC}_{ML}^{GF}-{KJC}_{ML}^{GF}\right)}M2+{GRF}_{V}^{GF}*\left({\;COP}_{ML}^{GF}-{AJC}_{ML}^{GF}\right)$$ (3)

Therefore, we considered the second component (M2) as the first term in Eq. 3, corresponding to the moment due to ${GRF}_{V}^{GF}$ with the lever arm as the lateral position of the knee relative to the ankle obtained from the horizontal projection of $L_{Shank}$ (Eq. 4; Figure 2C):

$${M2=GRF}_{V}^{GF}*\left({AJC}_{ML}^{GF}-{KJC}_{ML}^{GF}\right)={GRF}_{V}^{GF}*LShank*\sin\left(FPTA\right),$$ (4)

with FPTA , the frontal plane tibia angle.

Finally, the second term of Eq. 3 can be decomposed into a moment M3 with the lever arm corresponding to the medial position of COP relative to the ankle in the foot frame (FF) (in Eq. 5; Figure 2D), and another moment M4 with the lever arm corresponding to the medial position of COP relative to the ankle in the global frame caused by the foot progression angle (FPA) (Eq. 5; Figure 2E).

$${GRF}_{V}^{GF}*\left({\;COP}_{ML}^{GF}-{AJC}_{ML}^{GF}\right)=\overset{︷}{{{GRF}_{V}^{GF}*COP}_{ML}^{FF}*\cos\left(FPA\right)}M3+\overset{︷}{{GRF}_{V}^{GF}*{COP}_{AP}^{FF}*\sin\left(-FPA\right)}M4$$ (5)

The M3 could be negative, only if the COP is lateral to the foot axis (Figure 2C) but does not change the sign with the FPA. As shown in Figure 2F, if COP is medial to the foot axis, the positive contribution of the M3 (meaning that it increases the KAM) does not alter with toe-in or toe-out. In contrast, the negative/positive contribution of M4 depends on the FPA, with a negative contribution with toe-out (Figure 2G).

Depending on different GRFs and lever arms, each of these four components expresses a different mechanism modifying KAM. The moment M1 can vary with ${GRF}_{ML}^{GF}$ and the tibia angle in the sagittal plane. The moment M2 can vary with ${GRF}_{V}^{GF}$ and inclination of the tibia in the frontal plane (Figure 2C). A more lateral position of the knee relative to the ankle increases M2. The moment M3 depends on the mediolateral position of the COP in the foot frame multiplied by cosine (FPA). The more medial position of the COP increases the contribution of M3 and the lateral position of the COP with respect to the foot axis, which results in a negative M3 (Figure 2D). The moment M4 originates from the FPA and anterior position of the COP in the foot frame during the stance phase. It can be negative with toe-out or at heel strike when COP is posterior to the ankle joint center. The negative value of a component means that it generates a knee abduction moment.

### Statistical analysis

Quantitative variables were averaged over ten repetitions. The data were tested for normality with Shapiro–Wilk and Kolmogorov–Smirnov tests. Data were presented as the mean ± standard deviation (SD). The errors of the KAM features (i.e., first and second peaks and KAM impulse) estimated by dGRV were assessed against ID as the mean difference (bias) and standard deviation of difference (precision) over all ten averaged gait cycles. To evaluate the similarity of the dGRV model to ID in estimating the KAM profile during the stance phase of gait, we used a new formulation of the coefficient of multiple correlations (Ferrari et al., 2010) that is cleared from the between-gait cycle variability and assessed the inter-protocol similarity.

In order to evaluate the association between the KAM components and the brace or medial knee OA, we employed linear mixed-effects models. In each model, we adjusted for walking speed as a fixed effect and considered subject as a random effect. The statistical significance level was set to 0.05. Data were analyzed using Matlab (R2021b, Math Works, Natick, United States).

## Results

Out of 12 patients, five patients had both knees that met the inclusion criteria, so 24 limbs of 12 healthy volunteers and 17 limbs of 12 patients were included in the analysis (Table 1). This led to a total of 1,440 gait cycles for healthy volunteers (24 limbs × 3 speeds × 10 trials × 2 conditions) and 510 cycles for patients (17 limbs × 3 speeds × 10 trials × 1 condition).

**FIGURE 3:**
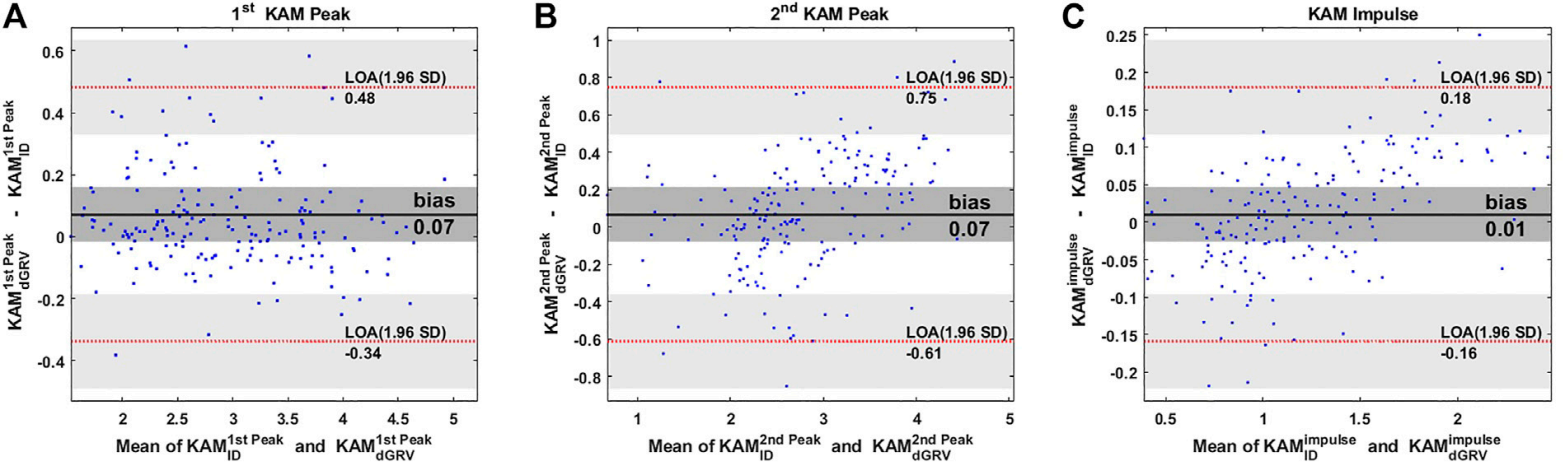
Bland–Altman plots comparing estimates of the **(A)** first peak, **(B)** second peak of the KAM, and **(C)** KAM impulse from the dGRV model compared to inverse dynamics (ID) averaged over ten gait cycles of all participants. The limit of agreement (LOA) is presented by the red dotted line and the bias by the black line. The gray zone indicates the 95% confidence interval for the bias and LOA (Giavarina, 2015).

The dGRV model predicted the whole KAM profile for all gait cycles of all subjects with a coefficient of multiple correlations of 0.98 ± 0.01. Compared to ID, bias ± precision was 0.07 ± 0.21 %Bw*Ht for the first peak, 0.07 ± 0.35 %Bw*Ht for the second peak, and 0.01 ± 0.08 %Bw*Ht*S for the KAM impulse (Figure 3; Table 2). The walking speed was altered from a self-selected speed when the subjects walked slowly and fast (Table 2). The bias and SD of the error of the dGRV model decreased at slower speeds in healthy volunteers (Table 2). This means that the dGRV model was more accurate and more precise at slower walking speeds where the movements were less accelerated. The dGRV model was more accurate in estimation of the first peak than in the second peak (both bias and precision were smaller in the first peak) (Table 2).

**TABLE 2:** Walking speed presented as mean ± SD and bias ± precision of the dGRV model compared to ID at different conditions (slow/self-selected/fast, with/without brace) and groups (healthy/patient).

	Conditions	Walking speed (m/s)	1st peak (%Bw*Ht)	2nd peak (%Bw*Ht)	KAM impulse (%Bw*Ht*S)
Healthy	Slow	0.96 ± 0.17	0.05 ± 0.11	0.06 ± 0.19	−0.01 ± 0.06
	Self-selected	1.28 ± 0.18	0.10 ± 0.20	0.03 ± 0.26	−0.00 ± 0.07
	Fast	1.60 ± 0.17	0.17 ± 0.30	0.17 ± 0.37	0.04 ± 0.08
	Slow/with brace	0.97 ± 0.11	0.05 ± 0.08	−0.09 ± 0.19	−0.05 ± 0.07
	Self-selected/with brace	1.32 ± 0.22	0.09 ± 0.23	−0.18 ± 0.34	−0.05 ± 0.08
	Fast/with brace	1.66 ± 0.26	0.12 ± 0.34	−0.17 ± 0.46	−0.04 ± 0.08
Patient	Slow	0.88 ± 0.10	0.00 ± 0.10	0.36 ± 0.15	0.11 ± 0.05
	Self-selected	1.14 ± 0.16	−0.02 ± 0.09	0.34 ± 0.15	0.08 ± 0.04
	Fast	1.48 ± 0.20	0.01 ± 0.11	0.35 ± 0.22	0.07 ± 0.06

**FIGURE 4:**
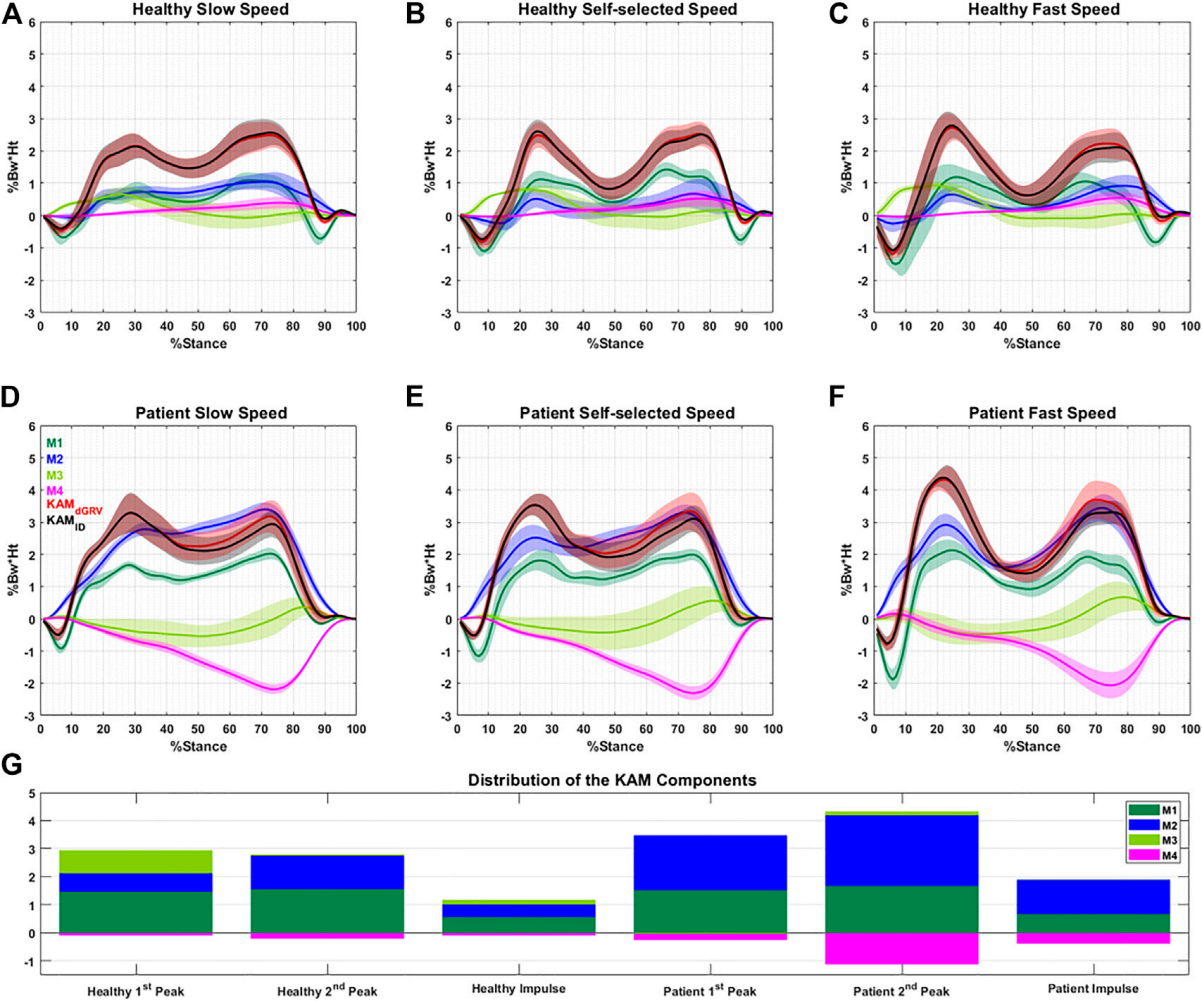
Profile of the four main components (M1–M4) of the normalized KAM (%Bw*Ht) with their summation (dGRV in red) during the stance phase for one typical healthy subject **(A–C)** and one typical patient **(D–F)** at different walking speeds. The profile of the KAM extracted using the inverse dynamic (ID) method is illustrated in black. Data are presented as the mean of 10 gait cycles at each condition bracketed with SD. **(G)** Average distribution of the components at the first, second peaks, and KAM impulse for all subjects. The negative value of a component means it generates knee abduction moment.

**FIGURE 5:**
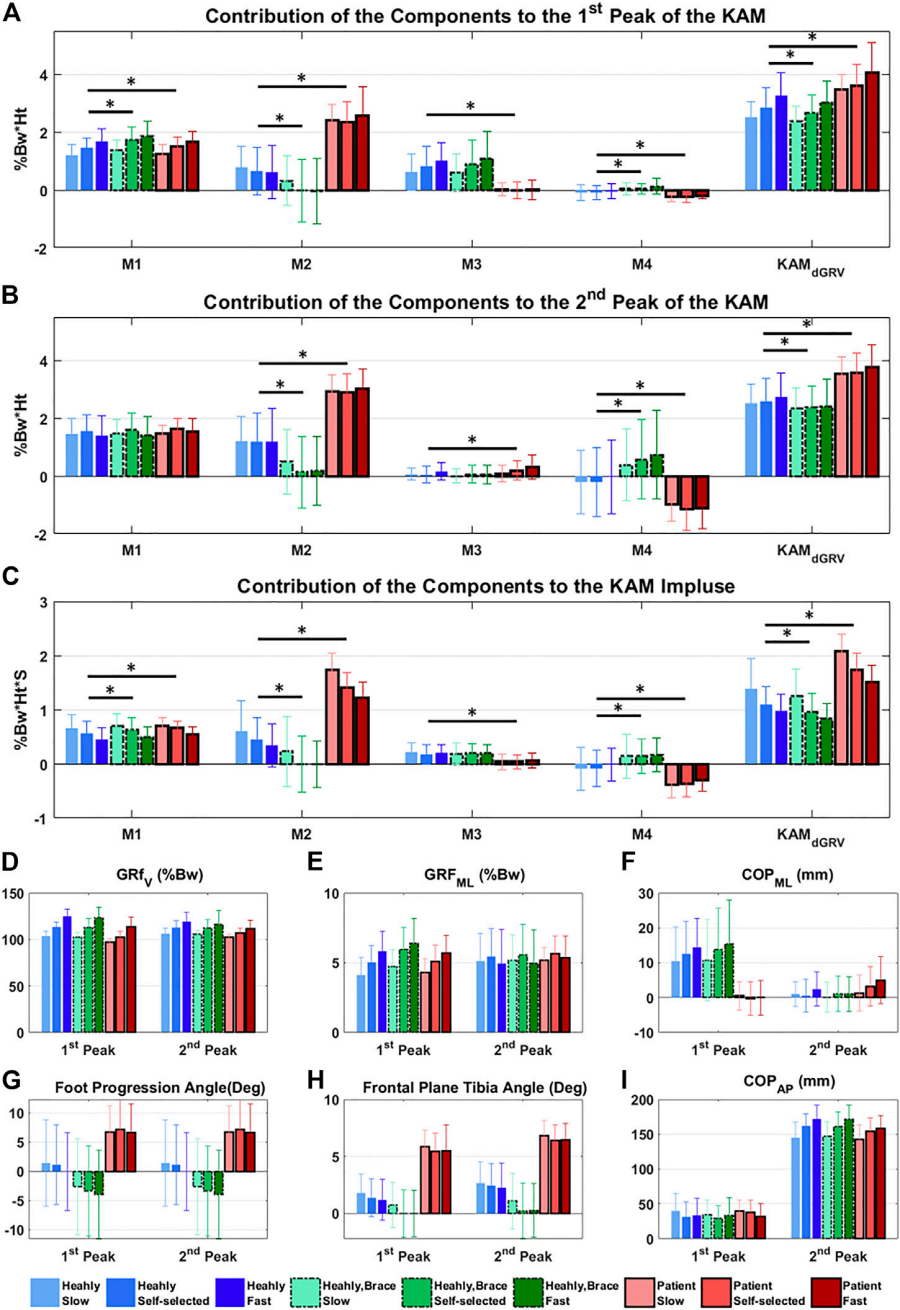
Contribution of the components of the KAM at different conditions (three walking speeds and with/without brace) to the **(A)** first peak, **(B)** second peak, and **(C)** impulse of the KAM. Significant difference extracted from the linear mixed-effects model adjusted for walking speed, between the healthy with/without brace and healthy/patient groups are illustrated by * (*p* < 0.05). **(D–I)** show biomechanical factors at the first and second peaks at different conditions.

The profiles of the four components of the KAM showed that some components had negative contributions to the KAM features (Figure 4), meaning that they generated a knee abduction moment. The positive (adduction)/negative (abduction) effect of M4 (which depends on the FPA) was similar in the first and second peaks but with a higher amplitude at the second peak. Although the M3 (dependent on the mediolateral position of COP) contributed to the first peak, it had a negligible contribution to the second peak with mean ± SD of 0.82 ± 0.69 vs. 0.05 ± 0.30 %Bw*Ht in healthy subjects (Figure 4G). Furthermore, its contribution to the first peak and KAM impulse in the patient group with varus deformity and medial knee OA were negligible (Figure 4; Supplementary Table S1). The linear mixed-effects model showed that compared to the healthy group, the contribution of M2 in the patient group increased drastically and formed the main component of the KAM, while the M4 decreased in all KAM features (Figure 4; Supplementary Table S1). The natural (not altered intentionally) FPA and frontal plane tibia angle were significantly greater in patients with varus deformity and medial knee OA by 6.5° [95% CI = (5.9, 7.1)], and 4.1° [3.9, 4.3], respectively (Figure 5; Supplementary Table S2). The brace significantly decreased the first peak by 0.21 %Bw*Ht [95% CI = (0.16, 0.270)], the second peak by 0.20 %Bw*Ht [0.13, 0.26], and the KAM impulse by 0.10 %Bw*Ht*S [0.06, 0.13] in the healthy group by reducing the M2 while increasing M4 (Figure 5; Supplementary Table S1). The brace decreased the FPA by 3.9° 95% CI = [3.6, 4.2]° and the frontal plane tibia angle by 1.1° [1.0, 1.2] at the first peak and 1.8° [1.6, 1.9] at the second peak (Figure 5; Supplementary Table S2). The increase in the walking speed increased the first peak significantly and decreased the KAM impulse in both the healthy (with/without brace) and patient groups with varus deformity and medial knee OA. No statistical difference was observed in the second peak with speed changes.

## Discussion

The main purpose of this study was to evaluate the accuracy of the dGRV model that identifies the KAM components. The secondary aim was to investigate each component’s contribution to main KAM features in participants where KAM is modified with a brace or varus deformity with medial knee OA. The dGRV model decomposes the lever arm of the GRF around the knee joint center into clinically understandable parameters related to gait modifications. The model showed a good performance in predicting KAM while highlighting the main components of KAM affected by altered health/disease and gait conditions (change in gait speed and use of a brace).

The first peak of KAM increased significantly with increasing speed in both healthy and patient groups. In the healthy group, this increase was mainly through increases in M1 (i.e., mediolateral GRF) and M3 (i.e., mediolateral COP position) and decrease in M2 (i.e., vertical GRF and the lateral knee position relative to ankle). The KAM impulse decreased with speed, while alterations in the second peak were not significant.

Our results revealed that M2 is the source of increased KAM in patients with varus deformity and medial knee OA (Figures 5A–C). Although the vertical GRF decreased in patients compared to healthy volunteers at a self-selected speed, the frontal plane tibia angle was increased and caused a larger M2 (Supplementary Tables S1, S2). Interestingly, the M3 was reduced significantly in patients with varus deformity and medial knee OA in the first peak and KAM impulse mainly due to more lateral positions of the COP in the foot frame (Figure 5F; Supplementary Table S2), which was in agreement with a previous study that observed more loading on the lateral aspect of the foot in patients with medial knee OA (Lidtke et al., 2010). The M4 was also decreased in all KAM features in patients with varus deformity and medial knee OA due to more toe-out gait. Although controversies exist on the effect of FPA, it is accepted that on average the toe-in gait reduces the first peak and toe-out reduces the second peak (Hunt et al., 2018; Wang et al., 2021). In the dGRV model, the toe-in leads to larger M4 in the first and second peaks. However, the secondary kinematic change of the toe-in gait is the reduction in the frontal plane tibia angle (Uhlrich et al., 2018) which, despite increasing the M4 and decreasing the M2, results in a reduction in the first peak. In the second peak, the M4 increases more than in the first peak with toe-in gait due to the progression of the COP toward the toes (Figures 2E, 4). Therefore, the subsequent reduction of M2 could not be sufficient to compensate for the increased M4. This leads to increased KAM at the second peak. So, the balance between these two components and their baseline values might be the source of divergence of the results in different subjects and studies and should be considered for personalized adjustment of the FPA. For example, a patient with a larger value in the first peak (compared to the second peak) with varus orientation of the tibia may benefit from toe-in gait, while a patient with a larger second peak and valgus tibia may not be a proper candidate for a toe-in modification. In the first case, toe-in gait can probably correct the alignment of the tibia toward valgus and reduce the lever arm of M2, thereby reducing M2’s contribution to both peaks. However, the toe-in would increase M4 in the second peak which may or may not (in the case of balancing with reduced M2) cause the second peak to rise from the smaller baseline, but ultimately, it could probably balance the two peaks and avoid excessive sudden loading on the medial side of the knee. The brace significantly reduced the frontal plane tibia angle and FPA, thus decreasing the M2 and raising the M4 in healthy individuals. The effect of the brace is, therefore, the opposite of the effect of varus deformity in patients with medial knee OA. This result suggests that probably subjects with more varus orientation of tibia and toe-out could better benefit from the brace, rather than subjects with a well-aligned tibia and naturally toe-in gait. No significant alteration in the mediolateral position of the COP was observed with the brace.

As illustrated in the dGRV model (Figure 2F; Eq. 5), for M3, the lever arm of the mediolateral position of the COP was affected by the amount of toe-out. This is in accordance with the results from two clinical studies (Sawada et al., 2016; Ulrich et al., 2020) that concluded subjects with smaller natural FPA are more likely to reduce their KAM using a lateral wedge insole.

Understanding the respective contribution of the KAM components is particularly important because, as shown in this study, they have different effects on the KAM features. Furthermore, it can help identify the source of the increased KAM and can help move forward toward a more personalized decision-making for the selection of gait modifications based on the main cause rather than a general strategy. Gerbrands et al. (2014) investigated the effect of five gait modifications on a healthy group and suggested an individual-based strategy should be selected because of the large variance in the results. Uhlrich et al. (2018, 2020) considered only one gait modification but showed that the personalized adjustment of the FPA is more effective in reducing the KAM than the non-personalized method. Therefore, identifying the components of the KAM could help diagnose the source of increased KAM in different subjects, and based on that, the most related gait modification could be devised.

Another benefit of this model is the simplicity and ease of measuring inputs, which could even be measured by wearable systems. The FPA and tibia inclination in the sagittal plane can be estimated by IMU sensors (Wouda et al., 2021), and the GRF and COP can be measured by instrumented insoles (He et al., 2019). Therefore, with fewer complications than ID, this model could serve as the basis for an analytical model to estimate KAM components with a wearable system or provide insights to feature selection to devise a machine learning model.

In this study, the gait modifications were limited to the gait speed for both groups and using the brace only for the healthy group, while the effect of the other modifications, such as toe-in, wider step width, and medial thrust, on the components of the KAM needs to be investigated. The brace was used only for healthy volunteers, while the response could be different in patients. To have more subjects, the healthy and patient groups were not completely matched specifically in terms of age and BMI, and it might limit the comparisons between them. The knee flexion moment was not investigated here, which could alter the loading condition in the knee joint. The small sample size may limit the robustness of the results.

## Conclusion

The dGRV model provides a simple analytical tool for clinicians to estimate the KAM with a coefficient of multiple correlations of 0.98 ± 0.01 compared to a sophisticated ID model. Second, it innovatively divided the KAM into four biomechanically interpretable components and proposed how a combination of tibia varus orientation, foot progression angle, and center of pressure can generate the KAM. This model also enabled comparisons of the KAM at the component-level instead of the general KAM that could help achieve a more personalized treatment. Finally, it identified how gait modifications can alter the components in healthy volunteers, and which components are responsible for increased KAM in patients with varus deformity and medial knee OA.

This decomposition allows a better understanding of the cause of the variable response of patients to treatments based on gait modifications and the divergence in the results between different subjects and studies. Identifying the contribution of the components to the KAM and their altered role in early and late stance could resolve the controversies raised by the conflicting results of previous clinical studies. Therefore, the dGRV model can address the increasing need for explaining the underlying mechanisms behind KAM and thus target more specific treatments based on personalized gait modifications. This could potentially improve the treatment efficacy on OA symptoms and progression. Further studies are needed with broader inclusion criteria to further test the clinical use of the personalized aspect of this model.

## Data Availability

The code for extracting the KAM components and raw data for one participant is freely available at Zenodo (doi: 10.5281/zenodo.7215806). The raw data of other participants are accessible from the corresponding author upon request.

## References

[B1] Baker R. (2020). Handbook of human motion. 10.1007/978-3-319-30808-1

[B2] Baker R. Leboeuf F. Reay J. Sangeux M. (2018). The conventional gait model - success and limitations. Handb. Hum. Motion 1–3, 489–508. 10.1007/978-3-319-14418-4_25

[B3] Baniasad M. Shojaee Fard M. Farahmand F. Aminian K. (2020). Can the ground reaction vector be an alternative to conventional gait model to estimate knee adduction moment? Gait Posture 81, 24–25. 10.1016/j.gaitpost.2020.07.035

[B4] Chehab E. F. Favre J. Erhart-Hledik J. Andriacchi T. (2014). Baseline knee adduction and flexion moments during walking are both associated with 5year cartilage changes in patients with medial knee osteoarthritis. Osteoarthr. Cartil. 22 (11), 1833–1839. 10.1016/j.joca.2014.08.009 PMC4369510 25211281

[B5] Cheung R. T. H. Ho K. Au I. An W. Zhang J. Chan Z. (2018). Immediate and short-term effects of gait retraining on the knee joint moments and symptoms in patients with early tibiofemoral joint osteoarthritis: A randomized controlled trial. Osteoarthr. Cartil. 26 (11), 1479–1486. 10.1016/J.JOCA.2018.07.011 30081075

[B6] Fantozzi S. Garofalo P. Cutti A. G. Stagni R. (2012). 3D joint moments in transfemoral and transtibial amputees: When is the ground reaction vector technique" an alternative to inverse dynamics? J. Mech. Med. Biol. 12 (4), 1254–1260. 10.1142/S0219519412004983

[B7] Ferrari A. Cutti A. G. Cappello A. (2010). A new formulation of the coefficient of multiple correlation to assess the similarity of waveforms measured synchronously by different motion analysis protocols. Gait Posture 31 (4), 540–542. 10.1016/j.gaitpost.2010.02.009 20303272

[B8] Ferrigno C. Wimmer M. A. Trombley R. M. Lundberg H. J. Shakoor N. Thorp L. E. (2016). A reduction in the knee adduction moment with medial thrust gait is associated with a medial shift in center of plantar pressure. Med. Eng. Phys. 38 (7), 615–621. 10.1016/J.MEDENGPHY.2016.03.008 27158051

[B9] Foroughi N. Smith R. Vanwanseele B. (2009). The association of external knee adduction moment with biomechanical variables in osteoarthritis: A systematic review. Knee 16 (5), 303–309. 10.1016/J.KNEE.2008.12.007 19321348

[B10] Gerbrands T. A. Pisters M. F. Vanwanseele B. (2014). Individual selection of gait retraining strategies is essential to optimally reduce medial knee load during gait. Clin. Biomech. 29 (7), 828–834. 10.1016/j.clinbiomech.2014.05.005 24917175

[B11] Giavarina D. (2015). Understanding bland altman analysis. Biochem. Med. Zagreb. 25 (2), 141–151. 10.11613/BM.2015.015 26110027 PMC4470095

[B12] He J. Lippmann K. Shakoor N. Ferrigno C. Wimmer M. A. (2019). Unsupervised gait retraining using a wireless pressure-detecting shoe insole. Gait Posture 70, 408–413. 10.1016/j.gaitpost.2019.03.021 30986588

[B13] Hunt M. A. Charlton J. Krowchuk N. Tse C. Hatfield G. (2018). A randomized controlled trial investigating clinical and biomechanical changes following toe-out gait modification for knee osteoarthritis. Osteoarthr. Cartil. 26, S16–S17. 10.1016/J.JOCA.2018.02.050 29709498

[B14] Hurwitz D. E. Ryals A. B. Case J. P. Block J. A. Andriacchi T. P. (2002). The knee adduction moment during gait in subjects with knee osteoarthritis is more closely correlated with static alignment than radiographic disease severity, toe out angle and pain. J. Orthop. Res. 20 (1), 101–107. 10.1016/S0736-0266(01)00081-X 11853076

[B15] Jenkyn T. R. Hunt M. A. Jones I. C. Giffin J. R. Birmingham T. B. (2008). Toe-out gait in patients with knee osteoarthritis partially transforms external knee adduction moment into flexion moment during early stance phase of gait: A tri-planar kinetic mechanism. J. Biomechanics 41 (2), 276–283. 10.1016/j.jbiomech.2007.09.015 18061197

[B16] Lidtke R. H. Muehleman C. Kwasny M. Block J. A. (2010). Foot center of pressure and medial knee osteoarthritis. J. Am. Podiatr. Med. Assoc. 100 (3), 178–184. 10.7547/1000178 20479447

[B17] Menger B. Kannenberg A. Petersen W. Zantop T. Rembitzki I. Stinus H. (2016). Effects of a novel foot–ankle orthosis in the non-operative treatment of unicompartmental knee osteoarthritis. Arch. Orthop. Trauma Surg. 136 (9), 1281–1287. 10.1007/s00402-016-2500-2 27393498 PMC4990629

[B18] Moyer R. F. Birmingham T. Bryant D. Giffin J. Marriott K. Leitch K. (2015). Biomechanical effects of valgus knee bracing: A systematic review and meta-analysis. Osteoarthr. Cartil. 23 (2), 178–188. 10.1016/j.joca.2014.11.018 25447975

[B19] Murray C. J. L. Vos T. Lozano R. Naghavi M. Flaxman A. D. Michaud C. (2012). Disability-adjusted life years (DALYs) for 291 diseases and injuries in 21 regions, 1990-2010: A systematic analysis for the global burden of disease study 2010. Lancet 380 (9859), 2197–2223. 10.1016/S0140-6736(12)61689-4 23245608

[B20] Ornetti P. Parratte S. Gossec L. Tavernier C. Argenson J. N. Roos E. (2008). Cross-cultural adaptation and validation of the French version of the Knee injury and Osteoarthritis Outcome Score (KOOS) in knee osteoarthritis patients. Osteoarthr. Cartil. 16 (4), 423–428. 10.1016/J.JOCA.2007.08.007 17905602

[B21] Pereira L. C. Ulrich B. Runhaar J. Bierma-Zeinstra S. Jolles B. Favre J. (2021). Effects of step width modifications on the ambulatory kinetics in patients with medial knee osteoarthritis. Osteoarthr. Cartil. 29, S11. 10.1016/J.JOCA.2021.05.020

[B22] Petersen W. Ellermann A. Henning J. Nehrer S. Rembitzki I. V. Fritz J. (2019). Non-operative treatment of unicompartmental osteoarthritis of the knee: A prospective randomized trial with two different braces—ankle–foot orthosis versus knee unloader brace. Arch. Orthop. Trauma Surg. 139 (2), 155–166. 10.1007/s00402-018-3040-8 30255369

[B23] Richards R. van den Noort J. C. Dekker J. Harlaar J. (2017). Gait retraining with real-time biofeedback to reduce knee adduction moment: Systematic review of effects and methods used. Archives Phys. Med. Rehabilitation 98 (1), 137–150. 10.1016/j.apmr.2016.07.006 27485366

[B24] Robbins S. M. K. Maly M. R. (2009). The effect of gait speed on the knee adduction moment depends on waveform summary measures. Gait Posture 30 (4), 543–546. 10.1016/j.gaitpost.2009.08.236 19748272

[B25] Sawada T. Tokuda K. Tanimoto K. Iwamoto Y. Ogata Y. Anan M. (2016). Foot alignments influence the effect of knee adduction moment with lateral wedge insoles during gait. Gait Posture 49, 451–456. 10.1016/j.gaitpost.2016.08.011 27541338

[B26] Shelburne K. B. Torry M. R. Steadman J. R. Pandy M. G. (2008). Effects of foot orthoses and valgus bracing on the knee adduction moment and medial joint load during gait. Clin. Biomech. (Bristol, Avon) 23 (6), 814–821. 10.1016/J.CLINBIOMECH.2008.02.005 18362043

[B27] Shull P. B. Lurie K. L. Cutkosky M. R. Besier T. F. (2011). Training multi-parameter gaits to reduce the knee adduction moment with data-driven models and haptic feedback. J. Biomechanics 44 (8), 1605–1609. 10.1016/j.jbiomech.2011.03.016 21459384

[B28] Uhlrich S. D. (2020). Personalization improves the biomechanical efficacy of foot progression angle modifications in individuals with medial knee osteoarthritis. medRxiv. 10.1101/2020.12.15.20248220 PMC9889103 36191434

[B29] Uhlrich S. D. Silder A. Beaupre G. S. Shull P. B. Delp S. L. (2018). Subject-specific toe-in or toe-out gait modifications reduce the larger knee adduction moment peak more than a non-personalized approach. J. Biomechanics 66, 103–110. 10.1016/j.jbiomech.2017.11.003 PMC5859947 29174534

[B30] Ulrich B. Hoffmann L. Jolles B. M. Favre J. (2020). Changes in ambulatory knee adduction moment with lateral wedge insoles differ with respect to the natural foot progression angle. J. Biomechanics 103, 109655. 10.1016/j.jbiomech.2020.109655 32057444

[B31] Wang S. Mo S. Chung R. C. K. Shull P. B. Ribeiro D. C. Cheung R. T. H. (2021). How foot progression angle Affects knee adduction moment and angular impulse in patients with and without medial knee osteoarthritis: A meta-analysis. Arthritis Care Res. Hob. 73 (12), 1763–1776. 10.1002/acr.24420 33242375

[B32] Wells R. P. (1981). The projection of the ground reaction force as a predictor of internal joint moments. Bull. Prosthet. Res. 18 (1), 15–19. 7332827

[B33] Wimmer M. A. Block J. A. (2007). Relationship between pain and medial knee joint loading in mild radiographic knee osteoarthritis. Arthritis Rheum. 57 (7), 1254–1260. Available at: https:// (Accessed July 30, 2021). 17907211 10.1002/art.22991

[B34] Wouda F. J. Jaspar S. L. J. O. Harlaar J. van Beijnum B. J. F. Veltink P. H. (2021). Foot progression angle estimation using a single foot-worn inertial sensor. J. Neuroeng. Rehabil. 18 (1), 37–10. 10.1186/S12984-021-00816-4 33596942 PMC7888122

[B35] Xing F. Lu B. Kuang M. j. Wang Y. Zhao Y. l. Zhao J. (2017). A systematic review and meta-analysis into the effect of lateral wedge arch support insoles for reducing knee joint load in patients with medial knee osteoarthritis. Med. (United States) 96 (24), e7168. 10.1097/MD.0000000000007168 PMC5478338 28614253

[B36] Xu J. Cao F. Zhan S. Ling M. Hu H. Shull P. B. (2020). Mapping-based dosage of gait modification selection for multi-parameter, subject-specific gait retraining. IEEE Access 8, 106354–106363. 10.1109/ACCESS.2020.2999473

