# Supplementary material for: Knee adduction moment decomposition: Toward better clinical decision-making
Source: Front Bioeng Biotechnol. 2022 Nov 18;10:1017711. doi: 10.3389/fbioe.2022.1017711 (PMC9715598; doi:10.3389/fbioe.2022.1017711)
Supplement: Supplementary file 1 [file Table1.DOCX]

# Supplementary

Table S1- Components of the knee adduction moment at 1^st^ and 2^nd^ peaks and impulse for healthy group walking with/without brace and patient group for three different speeds of walking. Data in the first three columns are presented as mean ± SD of the average of 10 gait cycles for each subject and walking condition. The last column reports the mean (95% confidence interval) of the difference between healthy with/without brace and healthy/patient, extracted from the linear mixed-effects models adjusted for walking speed. *: significant difference with self-selected speed within the same group (healthy/patient) and same condition (with/without brace). ᶤ: significant difference (p<0.05) between healthy with/without brace adjusted for walking speed. ꙳: significant difference (p<0.05) between healthy/patient adjusted for walking speed.

|  |  |  | **Slow**  **Mean±SD** | **Self-selected Mean±SD** | **Fast Mean±SD** | **Difference**  **Mean[95%CI]** |
| --- | --- | --- | --- | --- | --- | --- |
| **1^st^ Peak (% Bw*Ht)** | **M1** | **Healthy** | 1.20±0.36* | 1.46±0.35 | 1.69±0.42* |  |
|  |  | **Healthy (Brace)** | 1.39±0.35* | 1.75±0.45 | 1.85±0.52 | 0.21[0.17,0.25]ᶤ |
|  |  | **Patient** | 1.26±0.30* | 1.49±0.34 | 1.67±0.36* | 0.11[0.06,0.16]꙳ |
|  | **M2** | **Healthy** | 0.79±0.73 | 0.66±0.83 | 0.61±0.93 |  |
|  |  | **Healthy (Brace)** | 0.32±0.86* | -0.02±1.09 | -0.04±1.13 | -0.55[-0.61,-0.48]ᶤ |
|  |  | **Patient** | 2.42±0.56 | 2.35±0.71 | 2.57±1.00* | 1.76[1.67,1.85]꙳ |
|  | **M3** | **Healthy** | 0.62±0.62* | 0.82±0.69 | 1.02±0.63* |  |
|  |  | **Healthy (Brace)** | 0.61±0.64* | 0.89±0.84 | 1.09±0.94* | -0.03[-0.08,0.02] |
|  |  | **Patient** | 0.02±0.22 | -0.02±0.29 | 0.01±0.34 | -0.73[-0.78,-0.67]꙳ |
|  | **M4** | **Healthy** | -0.09±0.26 | -0.09±0.24 | -0.05±0.26 |  |
|  |  | **Healthy (Brace)** | 0.05±0.20 | 0.05±0.18 | 0.13±0.27* | 0.15[0.13,0.17]ᶤ |
|  |  | **Patient** | -0.24±0.17 | -0.23±0.19 | -0.19±0.12 | -0.16[-0.18,-0.13]꙳ |
|  | **KAM_dGRV_** | **Healthy** | 2.52±0.55* | 2.86±0.68 | 3.27±0.80* |  |
|  |  | **Healthy (Brace)** | 2.38±0.52* | 2.67±0.62 | 3.04±0.72* | -0.21[-0.27,-0.16]ᶤ |
|  |  | **Patient** | 3.47±0.53 | 3.60±0.76 | 4.06±1.06* | 0.98[0.89,1.07]꙳ |
| **2^nd^ Peak (% Bw*Ht)** | **M1** | **Healthy** | 1.46±0.54* | 1.56±0.56 | 1.41±0.67* |  |
|  |  | **Healthy (Brace)** | 1.48±0.49* | 1.59±0.60 | 1.42±0.65* | 0.04[-0.00,0.08] |
|  |  | **Patient** | 1.47±0.29* | 1.63±0.37 | 1.54±0.46 | 0.06[-0.01,0.12] |
|  | **M2** | **Healthy** | 1.21±0.86 | 1.19±0.99 | 1.19±1.15 |  |
|  |  | **Healthy (Brace)** | 0.50±1.11* | 0.14±1.24 | 0.19±1.20 | -0.86[-0.93,-0.79]ᶤ |
|  |  | **Patient** | 2.94±0.57 | 2.89±0.66 | 3.03±0.66* | 1.90[1.81,1.98]꙳ |
|  | **M3** | **Healthy** | 0.06±0.21 | 0.05±0.30 | 0.16±0.32* |  |
|  |  | **Healthy (Brace)** | 0.00±0.25 | 0.06±0.31 | 0.06±0.32 | -0.04[-0.09,0.00] |
|  |  | **Patient** | 0.08±0.30* | 0.20±0.34 | 0.31±0.43 | 0.11[0.05,0.16]꙳ |
|  | **M4** | **Healthy** | -0.21±1.09 | -0.21±1.19 | -0.02±1.28* |  |
|  |  | **Healthy (Brace)** | 0.39±1.24* | 0.58±1.38 | 0.75±1.53 | 0.67[0.61,0.72]ᶤ |
|  |  | **Patient** | -0.97±0.59* | -1.13±0.74 | -1.12±0.72 | -1.05[-1.15,-0.95]꙳ |
|  | **KAM_dGRV_** | **Healthy** | 2.52±0.68 | 2.59±0.79 | 2.74±0.84* |  |
|  |  | **Healthy (Brace)** | 2.36±0.71 | 2.37±0.76 | 2.41±0.93 | -0.20[-0.26,-0.13]ᶤ |
|  |  | **Patient** | 3.53±0.60 | 3.59±0.67 | 3.76±0.79* | 1.01[0.91,1.10]꙳ |
| **KAM Impulse (% Bw*Ht*S)** | **M1** | **Healthy** | 0.65±0.24* | 0.56±0.22 | 0.45±0.22* |  |
|  |  | **Healthy (Brace)** | 0.70±0.22* | 0.62±0.22 | 0.49±0.19* | 0.07[0.05,0.09]ᶤ |
|  |  | **Patient** | 0.69±0.16* | 0.66±0.13 | 0.54±0.14* | 0.06[0.03,0.08]꙳ |
|  | **M2** | **Healthy** | 0.60±0.56* | 0.45±0.41 | 0.34±0.40* |  |
|  |  | **Healthy (Brace)** | 0.22±0.64* | -0.01±0.52 | -0.01±0.43 | -0.35[-0.39,-0.32]ᶤ |
|  |  | **Patient** | 1.73±0.32* | 1.40±0.29 | 1.22±0.28* | 0.97[0.93,1.01]꙳ |
|  | **M3** | **Healthy** | 0.22±0.18* | 0.17±0.18 | 0.20±0.14 |  |
|  |  | **Healthy (Brace)** | 0.18±0.20 | 0.19±0.18 | 0.19±0.17 | -0.02[-0.04,0.00] |
|  |  | **Patient** | 0.04±0.14 | 0.04±0.13 | 0.05±0.14 | -0.15[-0.18,-0.12]꙳ |
|  | **M4** | **Healthy** | -0.09±0.39 | -0.09±0.34 | -0.01±0.30* |  |
|  |  | **Healthy (Brace)** | 0.14±0.41 | 0.15±0.32 | 0.16±0.32 | 0.21[0.19,0.22]ᶤ |
|  |  | **Patient** | -0.40±0.24 | -0.37±0.25 | -0.31±0.20* | -0.32[-0.35,-0.29]꙳ |
|  | **KAM_dGRV_** | **Healthy** | 1.38±0.55* | 1.09±0.33 | 0.98±0.30* |  |
|  |  | **Healthy (Brace)** | 1.24±0.50* | 0.95±0.36 | 0.83±0.28* | -0.10[-0.13,-0.06]ᶤ |
|  |  | **Patient** | 2.07±0.31* | 1.73±0.32 | 1.51±0.32* | 0.55[0.50,0.60]꙳ |

Table S2- Biomechanical factors at 1^st^ and 2^nd^ peak. Data in the first three columns are presented as mean ±SD. The last column reports the mean (95% confidence interval) of the difference between healthy with/without brace and healthy/patient, extracted from the linear mixed-effects models adjusted for walking speed.

GRFv: vertical ground reaction force, GRFml: mediolateral ground reaction force, COPml: mediolateral position of center of pressure in foot frame, FPTA: Frontal plane tibia angle, FPA: Foot progression angle.

*: significant difference with self-selected speed within the same group (healthy/patient) and same condition (with/without brace). ᶤ: significant difference (p<0.05) between healthy with/without brace adjusted for walking speed. ꙳: significant difference (p<0.05) between healthy/patient adjusted for walking speed.

|  |  |  | **Slow**  **Mean±SD** | **Self-selected Mean±SD** | **Fast**  **Mean±SD** | **Difference**  **Mean[95%CI]** |
| --- | --- | --- | --- | --- | --- | --- |
| **1st Peak** | **GRFv (%Bw)** | **Healthy** | 103.8±5.5* | 113.6±5.3 | 125.0±7.9* |  |
|  |  | **Healthy(Brace)** | 102.8±5.0* | 113.0±10.1 | 123.3±11.5* | -2.1[-2.9,-1.2]ᶤ |
|  |  | **Patient** | 97.5±3.7* | 102.6±6.5 | 113.4±10.7* | -7.3[-8.3,-6.3]꙳ |
|  | **GRFml (%Bw)** | **Healthy** | 4.1±1.3* | 5.0±1.2 | 5.8±1.5* |  |
|  |  | **Healthy(Brace)** | 4.8±1.2* | 6.0±1.6 | 6.4±1.8* | 0.7[0.5,0.8]ᶤ |
|  |  | **Patient** | 4.3±1.0* | 5.1±1.2 | 5.7±1.2* | 0.3[0.1,0.5]꙳ |
|  | **COPml**  **(mm)** | **Healthy** | 10.4±9.8* | 12.6±9.4 | 14.4±8.4* |  |
|  |  | **Healthy(Brace)** | 10.7±11.6* | 13.8±11.9 | 15.3±12.7* | -0.3[-1.1,0.5] |
|  |  | **Patient** | 0.5±4.1 | -0.3±4.9 | 0.0±5.0 | -11.4[-12.3,-10.5]꙳ |
|  | **FPA**  **(Deg)** | **Healthy** | 1.4±7.4 | 1.2±6.8 | -0.1±6.7* |  |
|  |  | **Healthy(Brace)** | -2.6±8.2* | -3.4±7.7 | -3.9±7.6 | -3.9[-4.2,-3.6]ᶤ |
|  |  | **Patient** | 6.8±4.4 | 7.2±5.1 | 6.7±4.9 | 6.5[5.9,7.1]꙳ |
|  | **FPTA**  **(Deg)** | **Healthy** | 1.8±1.7* | 1.4±1.7 | 1.2±1.8 |  |
|  |  | **Healthy(Brace)** | 0.7±2.0* | -0.0±2.1 | 0.0±2.0 | -1.1[-1.2,-1.0]ᶤ |
|  |  | **Patient** | 5.9±1.4* | 5.4±1.6 | 5.5±2.3 | 4.1[3.9,4.3]꙳ |
| **2nd Peak** | **GRFv (%Bw)** | **Healthy** | 106.3±5.9* | 112.9±7.3 | 119.3±9.9* |  |
|  |  | **Healthy(Brace)** | 105.6±4.3* | 112.2±9.1 | 116.0±15.4 | -1.4[-2.4,-0.4]ᶤ |
|  |  | **Patient** | 102.5±3.3* | 106.8±5.7 | 111.8±9.0* | -4.0[-5.0,-3.0]꙳ |
|  | **GRFml (%Bw)** | **Healthy** | 5.1±2.0* | 5.5±2.0 | 4.9±2.4* |  |
|  |  | **Healthy(Brace)** | 5.2±1.8* | 5.6±2.2 | 5.0±2.4* | 0.1[-0.0,0.3] |
|  |  | **Patient** | 5.2±0.9* | 5.7±1.2 | 5.4±1.6 | 0.1[-0.1,0.4] |
|  | **COPml**  **(mm)** | **Healthy** | 1.0±3.6 | 0.6±4.7 | 2.4±4.9* |  |
|  |  | **Healthy(Brace)** | 0.1±4.3 | 1.1±5.1 | 1.1±5.0 | -0.5[-1.3,0.2] |
|  |  | **Patient** | 1.4±5.1* | 3.3±5.7 | 5.0±6.7 | 1.8[0.9,2.8]꙳ |
|  | **FPTA**  **(Deg)** | **Healthy** | 2.7±1.9 | 2.5±1.9 | 2.3±2.2 |  |
|  |  | **Healthy(Brace)** | 1.1±2.4* | 0.2±2.5 | 0.3±2.3 | -1.8[-1.9,-1.6]ᶤ |
|  |  | **Patient** | 6.8±1.3* | 6.4±1.4 | 6.4±1.4 | 4.3[4.2,4.5]꙳ |
